# Mealybug Chromosome Cycle as a Paradigm of Epigenetics

**DOI:** 10.1155/2012/867390

**Published:** 2012-04-08

**Authors:** Giorgio Prantera, Silvia Bongiorni

**Affiliations:** ^1^Department of Ecology and Biology, University of Tuscia, Via San Camillo de Lellis, 01100 Viterbo, Italy; ^2^Department for Innovation in Biological, Agro-food and Forest systems, University of Tuscia, Via San Camillo de Lellis, 01100 Viterbo, Italy

## Abstract

Recently, epigenetics has had an ever-growing impact on research not only for its intrinsic interest but also because it has been implied in biological phenomena, such as tumor emergence and progression. The first epigenetic phenomenon to be described in the early 1960s was chromosome imprinting in some insect species (*sciaridae* and *coccoideae*). Here, we discuss recent experimental results to dissect the phenomenon of imprinted facultative heterochromatinization in Lecanoid coccids (mealybugs). In these insect species, the entire paternally derived haploid chromosome set becomes heterochromatic during embryogenesis in males. We describe the role of known epigenetic marks, such as DNA methylation and histone modifications, in this phenomenon. We then discuss the models proposed to explain the noncanonical chromosome cycle of these species.

## 1. Epigenetics

The first appearance of the term epigenetics can be ascribed to Conrad Waddington, who stated in 1942 that “*epigenetics is the branch of biology which studies the causal interactions between genes and their products, which bring the phenotype into being*” [1]. In the modern view, epigenetics encompasses all those hereditary (genetic) phenomena not depending on the DNA sequence itself but on some functionally relevant molecular signatures which are imposed over the sequence (“*epi*” in Greek means “over”). All the systems involved in gene expression regulation are based on interactions between proteins and DNA. Some mechanisms inhibit or activate the expression of a single gene, acting on the promoter region, and thus reflect the structural organization of the gene itself (gene regulation). However, the *epi*-genetic systems can regulate phenotypic expression regardless of the gene sequence and are transmitted from one cell generation to the next one or from the parents to their progeny. These systems modulate the functional behavior of chromosomal regions, entire chromosomes, or even whole sets of chromosomes [2]. According to Denise Barlow “*epigenetics has always been all the weird and wonderful things that cannot be explained by genetics.*” Epigenetic phenomena occur in all the kingdoms from yeast to metazoans and plants. Some are limited to just one or few species. For example, RIP (rearrangement induced premeiotically) [3] and MIP (methylation induced premeiotically) [4] were reported in fungi, where they seem to protect the genome from transposable elements. The term paramutation, on the other hand, was coined to describe a heritable change in gene expression of an allele imposed by the presence of another specific allele, which occurs only in plants [5]. Paramutation seems to require the physical interaction between the two homologous alleles [6], as does the quite similar transvection phenomenon described in *Drosophila *by Lewis in 1954 [7]. Other phenomena are universal, at least in eukaryotes. These include, for example, the double-stranded RNA-mediated posttranscriptional gene silencing (PTGS).

Classical genetics has always considered the two parental copies of a gene functionally equivalent in determining the offspring phenotype, regardless of their origin. Genomic imprinting identifies, instead, the epigenetic process by which specific genes, single chromosomes, or entire haploid chromosome sets exhibit a differential functional behaviour, that is, dependent upon their parental origin [8–10]. The first evidences for the existence of genomic imprinting (and indeed the first use of this term in a genetic sense) came from the early works by Helen Crouse, in 1960s, on the fungus gnat *Sciara coprophila *[11, 12], and the subsequent studies on *Coccidae* [13, 14], showing that reciprocal crosses are not always equivalent (reviewed in [15, 16]). Nonetheless, those findings were seen as curiosities in their time, until imprinting evidence was uncovered in the mouse, in mid-1980s [17, 18].

A strong impetus to genomic imprinting studies came from the demonstration that failure of imprinting is responsible for severe syndromes in humans (reviewed in [19, 20]). For example, some human syndromes are caused by the transmission of both homologs from a single parent (uniparental disomy) [21].

**Figure 1:**
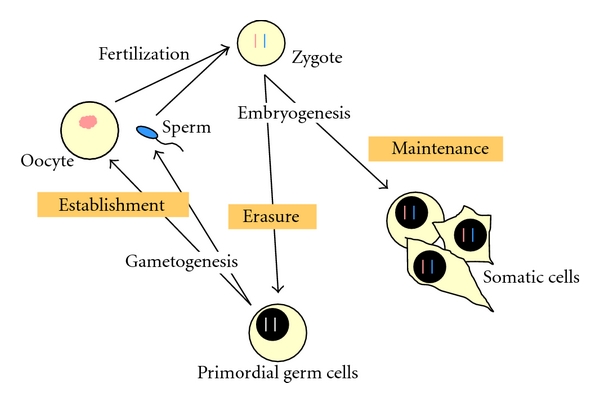
The “life cycle” of parental imprinting. The elaboration of the parent-of-origin-specific epigenetic information during animal development is achieved in three steps, namely, establishment, maintenance, and erasure. The paternal genome is illustrated in blue, the maternal genome in pink.

The elaboration of the parent-of-origin-specific epigenetic information proceeds through three steps, namely, establishment, maintenance, and erasure (Figure 1) (reviewed in [22, 23]). During gametogenesis a genome-wide erasure of the parent-specific epigenetic “marks” occurs, followed by the establishment of the signatures specific for each sex. After fertilization, the differential epigenetic marks, carried by the two parental pronuclei, are maintained and faithfully transmitted through the subsequent mitotic divisions during development. The imprinting marks specific to each parental allele are then “read” by the cellular machinery and translated into a differential, parent-of-origin-specific functional behavior. Genomic imprinting represents a paradigmatic example of epigenetic regulation, found not only in insects and mammals but also in yeast and plants [24, 25].

Hereafter we will describe the unusual chromosome system of the Lecanoid coccids (mealybugs), and the molecular machinery which is used by males of these insects to perform one of the most striking epigenetic phenomena: the imprinted facultative heterochromatinization of the entire paternal haploid chromosome set.

## 2. The Mealybug Chromosome System

Coccid insects are very small, most species are less than one centimeter in length. This group of Hemiptera exhibits “sexual dimorphism.” The body shape of females is globose and flattened, with the fusion of the head to thorax. Other segmental boundaries are often not clearly visible. Females are always wingless and frequently neotenic; they are covered with protective secretions such as wax, lacquer or, silk. Males are much smaller than females and have an elongated body with wings. 

**Figure 2:**
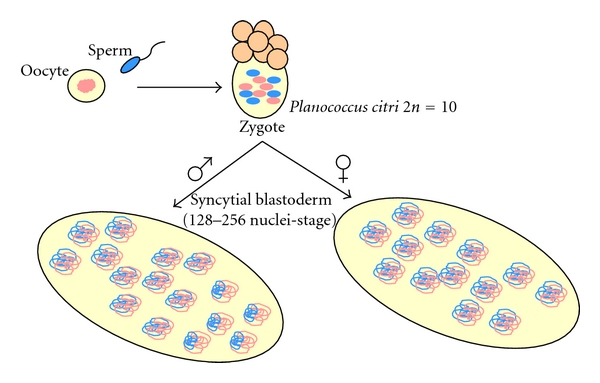
Male and female *P. citri *embryos at syncytial blastoderm (7th mitotic division, 128–256-nuclei stage). In the male embryo (left), it is possible to observe different stages of heterochromatinization. Heterochromatinization proceeds as a wave from one pole (bottom right) of the embryo, where nuclei show a fully developed chromocenter, toward the other one (top left), where nuclei still lack a chromocenter. Heterochromatinization selectively affects the paternal chromosomes (blue). In the female embryo (right), paternal and maternal (pink) chromosomes present the same degree of compaction and remain active.

Coccid species are identified based on male and female morphology, as well as on the karyotype. At the beginning of last century, two large groups were identified on the basis of morphological criteria the Margaroididae and the Lecano-diaspidoidae [26]. The karyocytological analysis confirmed the validity of this subdivision [27]. The Margaroididae retain the XX-XO mechanism of sex determination. In the Lecano-Diaspidoidae there are no differentiated sex chromosomes but these species possess a very complex and intriguing chromosome system. In the male line of Diaspidoids the whole paternal chromosome complement is discarded from midcleavage embryo cells; while in Lecanoid (mealybug) males, the whole paternally derived chromosome set undergoes heterochromatinization and the males become functionally haploid, a condition known as parahaploidy (Figure 2). After fertilization, all the embryo chromosomes are euchromatic. However, in female embryos all the chromosomes retain the euchromatic state, whereas in embryos destined to develop into males, the whole haploid set of paternal chromosomes becomes heterochromatic after the 7th cleavage division (Figure 2) [28]. This implies that, at least in males, the parental origin of the two chromosome sets must be distinguishable until blastoderm stage, when the heterochromatinization process specifically acts upon the paternally derived chromosomes. The process of heterochromatinization may thus be fruitful in the investigation of the behavior of epigenetic marks before and across the onset of heterochromatinization (see Section 3 for details). 

The mealybug chromosome system exhibits the characteristics of a genuine imprinting phenomenon [29]. The imprint is established in the gametes, maintained through the embryonic and adult somatic cell divisions, and erased in the germline (Figure 1). In somatic cells, the heterochromatic paternal chromosomes cluster and form a chromocenter that makes it very easy to distinguish male from female embryos. The chromocenter is noticeable in the nuclei of most tissues except the Malpighian tubules and the gut, where facultative heterochromatin reverts to a euchromatic state [30, 31]. The maternal euchromatic chromosomes are always distinguishable from the paternal ones until metaphase, when they too reach a high degree of condensation. Based on these features, paternal chromosome inactivation in male mealybugs represents, together with X-chromosome inactivation in female mammals, the most clear and large-scale example of facultative heterochromatinization. Facultative heterochromatinization may be defined as the developmentally regulated and tissue-specific *cis*-spreading of a heterochromatic state onto a euchromatic region, with a remodeling of the chromatin conformation that eventually leads to inactivation of all the genes it harbors. Distinct from constitutive heterochromatin, facultative heterochromatin is not composed of specific DNA sequences and in general involves only one of the two homologous sites; in these aspects it represents a true epigenetic phenomenon. 

The paternal origin of the heterochromatic set was established by Brown and Nelson-Rees (1961) [32]. These authors irradiated *Planococcus citri *males with X-rays prior to mating and then scrutinized their male offspring. Due to their holocentric nature, the chromosome fragments are not lost during paternal spermatogenesis and embryo development so the authors could demonstrate that the radiation-induced chromosomal aberrations were present only in heterochromatic haploid set of the sons. In contrast, in the male offspring of X-ray-treated females, only the euchromatic chromosomes were damaged. Using an analogous strategy, the same authors demonstrated the genetic inactivity of the heterochromatic set [32]. The parahaploid male progeny of X-ray-treated males exhibited normal vitality, whereas the survival of the diploid daughters decreased with increasing X-ray dose. This apparent paradox can be easily explained if one considers that, in the sons, any paternally-transmitted mutation was harbored by heterochromatic chromosomes and hence was not expressed, while in female progeny, any dominant lethal mutation was expressed. Nevertheless, the heterochromatic haploid chromosome set is not completely genetically inert in males since at least three different effects were found that could be ascribed to some residual activity of the paternal genome. First, the survival of male offspring of heavily X-ray-treated males (60.000 to 90.000 rep) depended on the amount of heterochromatic material, since the loss of heterochromatic fragments reduced the vitality [33]. The second effect was related to fertility: 100% of the male offspring of irradiated males (30,000 rep) survived, but a large percentage was sterile, and the frequency of sterile individuals increased with the radiation dose [33]. The third effect can be deduced by the observation that, in the male progeny of interspecific crosses, the heterochromatic set from one species could not be substituted for that (of an equivalent amount) of another [34]. Moreover, the activity of heterochromatic rDNA loci was demonstrated by the observation that in male cells ribosomal genes located on heterochromatic chromosomes were associated with nucleoli and nascent rRNA [35]. 

**Figure 3:**
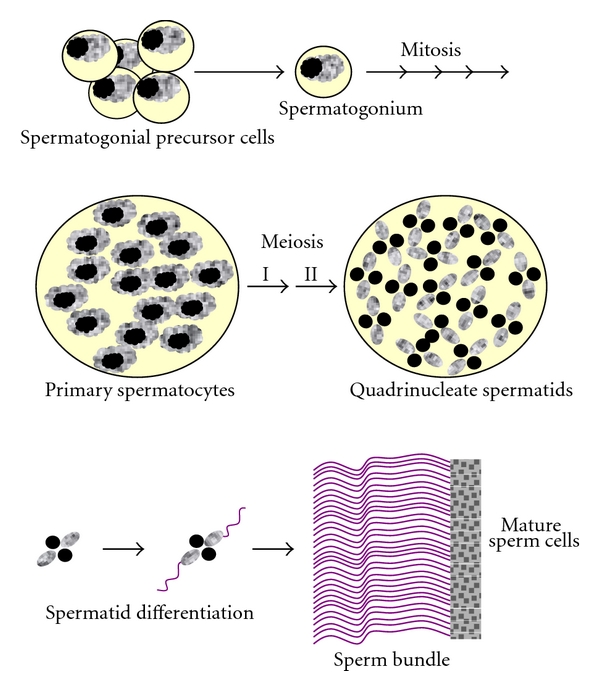
*P. citri *spermatogenesis. Each spermatogonial precursor cell produces a cluster of synchronously dividing spermatogonia, and after four mitotic divisions a cyst of 16 primary spermatocytes is obtained. Primary spermatocytes undergo an inverted type of meiosis, characterized by a nonindependent assortment. Meiosis produces a quadrinucleate cell with 2 elongated spermatids containing only the euchromatic chromosomes (gray staining), and 2 picnotic spermatids containing only the heterochromatic chromosomes (dark staining). Only euchromatic spermatids differentiate into 32 mature sperms.

**Figure 4:**
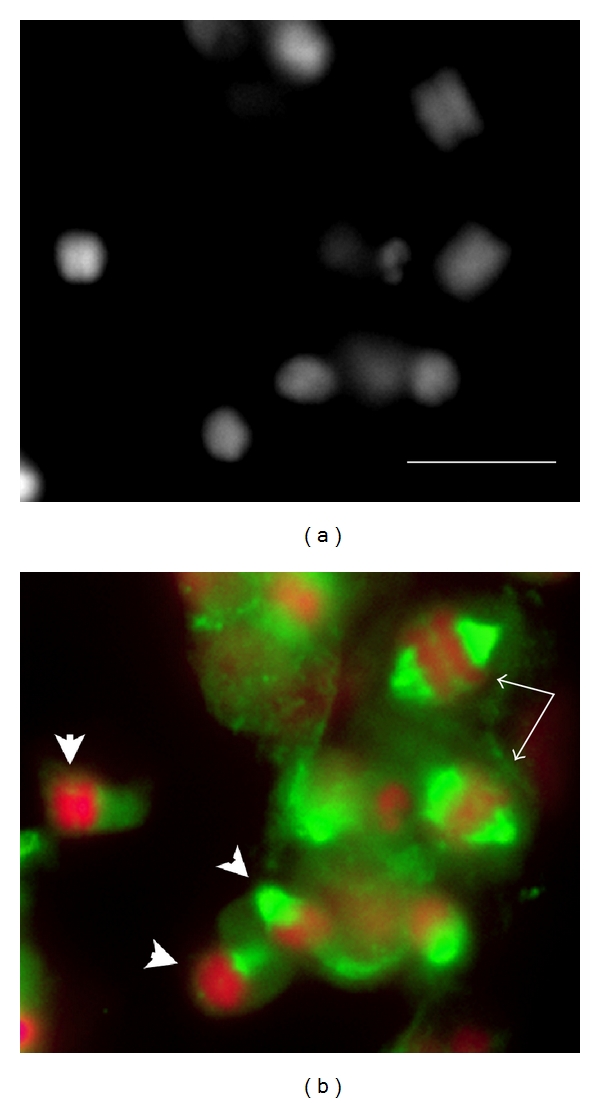
*P. citri *male meiosis. Meiotic sector from a testis of a third instar male. (a) DAPI-staining; (b) the same tissue patch, stained for DNA (pseudocolored in red) and for meiotic spindle, by an antibody against *α*-tubulin (pseudocolored in green). The DNA staining shows the metaphase I (arrow) and metaphase II (arrowheads) plates that can be distinguished on the basis of their different sizes (see Bongiorni et al., 2004, [37]). The meiotic spindle immunostainingstaining shows that meiosis II metaphase plates are associated with a monopolar spindle. Bar represents 10 *μ*m.

In mealybugs, the meiosis is atypical since the meiotic divisions are inverted. In male and female mealybugs, the first meiotic division is equational, with separation of sister chromatids, while the second one is reductional with segregation of the homologs (*Inverted meiosis*) [36, 37]. However, in females the remaining meiotic events are canonical, since homologous chromosomes undergo crossing over and independent assortment. During male meiosis, each spermatogonial precursor cell produces a cluster of synchronously dividing spermatogonia. Each spermatogonium divides four times to produce a cyst of 16 primary spermatocytes which then undergo the two meiotic divisions (Figure 3) [30]. The reductional second meiotic division is characterized by a nonindependent assortment of chromosomes, with the maternal euchromatic set segregating from the paternal heterochromatic one through a monopolar spindle (Figure 4) [37]. The two meiotic divisions thus generate a quadrinucleate spermatid with two nuclei containing the maternally derived euchromatic chromosomes and other two nuclei containing the paternally derived heterochromatic ones. Only spermatids containing the euchromatic chromosomes differentiate into sperm, while the heterochromatic products fail to form mature sperm and slowly degenerate *in situ*. The final result is the formation of a 64-nuclei cyst, where only the 32 “euchromatic” spermatids start the elongation process that ends with the production of 32 mature sperm (Figure 3). As a consequence of this extreme meiotic drive, only the maternally derived euchromatic chromosomes are transmitted to the progeny.

In summary, mealybug males exhibit not only two relevant epigenetic phenomena, chromosome, imprinting, and facultative heterochromatinization on a genome-wide scale, but also a dramatic deviation from canonical meiosis represented by inverted meiosis, nonindependent chromosome assortment and extreme meiotic drive.

## 3. The Mechanisms of Imprinted Facultative Heterochromatization in Mealybug

We carried out an extensive scrutiny of the epigenetic mechanisms in the mealybug *P. citri *and found that the machinery underpinning imprinted facultative heterochromatinization involves HP1-like and HP2-like proteins, as well as specific posttranslational histone modifications [28, 38–42]. Chromatin remodeling events have been commonly indicated as a mechanism by which eukaryotic cells regulate most of the epigenetic phenomena (reviewed in [43]), though the relevance of histone modifications as the carrier of epigenetic memory has been questioned [44]. Chromatin remodeling involves the interplay of many different posttranslational modifications of histones and of a number of nonhistone proteins. Histone modifications play a central role in the regulation of gene expression, and this led some authors to postulate the existence of a “histone code” as a regulatory code modulating the potentialities of the genetic code [45, 46]. Though the crosstalk of histone modifications does actually influence chromatin function, their combinations probably do not identify a true code.

**Figure 5:**
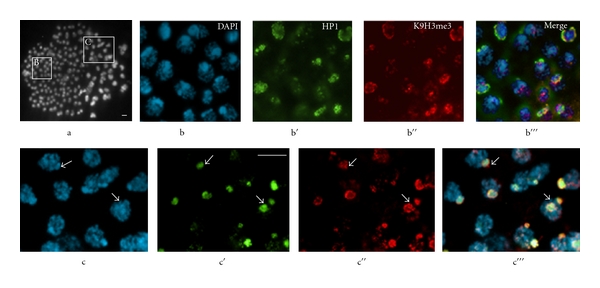
Pattern of epigenetic marks in *P. citri *cleavage embryos. Localization of C1A9 (anti-HP1) and anti-K9H3me3 antibodies to nuclei in midcleavage embryos (128–256-nuclei embryos) undergoing facultative heterochromatinization. (a) Whole embryo: the wave of facultative heterochromatinization is spreading from the bottom left corner toward the top right corner. Boxed area (B) shows nuclei that have completed heterochromatinization and contain DAPI-positive chromocenters (see magnified image in b), whereas boxed area (C) shows nuclei still undergoing heterochromatinization, many of which have no overt DAPI-positive chromocenters (arrows in c). The nuclei in b are labeled with the anti-HP1 antibody (b**′**) and the anti-K9H3me3 antiserum (b**′′**). The merged image in b**′′′**shows colocalization of DAPI-positive chromocenter, HP1 and K9H3me3 staining. The DAPI-stained nuclei in c were stained with anti-HP1 antibody (c**′**) and the anti-K9H3me3 antiserum (c**′′**). Although the pattern of K9H3me3 staining is more spread out in these nuclei compared with those that have completed heterochromatinization (compare c**′′**to b**′′**) the merged image in c**′′′**shows that the K9H3me3 pattern largely colocalizes with HP1 staining. C1A9 antibody recognizes the *P. citri *HP1-like, PCHET2. Bars, 10 *μ*m. Reprinted from Bongiorni et al. 2007 [40].

**Figure 6:**
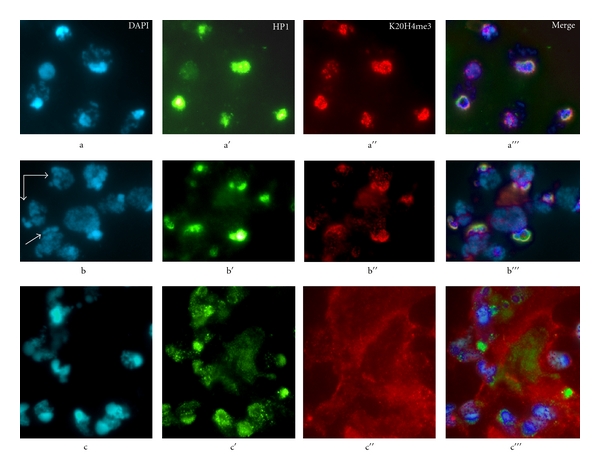
Localization of K20H4me3 in *P. citri *nuclei undergoing either facultative heterochromatinization or developmental de-heterochromatinization. (a) DAPI-stained nuclei from a midcleavage embryo (128–256-nuclei embryo) that underwent facultative heterochromatinization. A clear DAPI-stained chromocenter can be seen in each nucleus. The same nuclei were labeled with C1A9 (anti-HP1) antibody (a′) and with the anti-K20H4me3 antiserum (a′′); the merged image in a′′′ shows coincidence of DAPI-positive chromocenters with HP1 and K20H4me3 staining. The nuclei in b are from another area of the same embryo that has yet to complete heterochromatinization and several have no overt DAPI-positive chromocenters. The DAPI-stained nuclei in b were simultaneously stained by anti-HP1 antibody (b′) and by the anti-K20H4me3 antiserum (b′′). Whereas the K20H4me3 staining is more dispersed in these nuclei compared with those that have completed heterochromatinization (compare b′′ to a′′), the merged image (b′′′) shows that the K20H4me3 pattern largely colocalizes with HP1 staining. (c) DAPI-stained nuclei from cells of adult tissues that undergo developmental reversal of heterochromatinization. HP1 staining is dispersed and has a grainy appearance over the nuclei (c′) that, instead, lack any K20H4me3 staining which is rather dispersed over the cytoplasm (c′′). (c′′′) Merged image. Bars, 10 *μ*m. Reprinted from Bongiorni et al. 2007 [40].

The interplay between the heterochromatin protein HP1 [47] and the lysine 9 trimethylated isoform of the histone H3 (K9H3me3) has been shown to be pivotal for the assembly of silent chromatin domains [48–50]. The human (SUV39H1), the murine (Suv39h), and the Drosophila (SU(VAR)3-9) histone methyltransferases (HMTases), that selectively di- and trimethylate the histone H3 at lysine 9, generate a binding site for HP1 family proteins [48–50]. Moreover, in mammals, yeast, and Drosophila, it has been also shown that HP1 is, in turn, associated with the K9H3 HMTase, suggesting a self-maintenance model for the propagation of heterochromatic domains in native chromatin, that may well be responsible for epigenetic memory [51–54]. Facultative heterochromatinization in the nuclei of male mealybugs does not occur simultaneously in all cells of the 7th cleavage embryo but takes place as a wave, beginning at one end of the embryo and spreading to the other (Figure 2) [28]. HP1-like distribution in *P. citri *embryos was investigated using an antibody against *Drosophila *HP1 (C1A9 antibody [47]) [28]; this antibody recognized a protein of similar mass (29 kDa) which shared the *Drosophila *HP1 epitope [10, 28]. The establishment of a well-formed chromocenter in male embryo nuclei was preceded by the appearance of aggregates of HP1-like immunostaining that then continued to decorate the male-specific heterochromatin [28]. These results led us to hypothesize that the *P. citri *HP1-like might play a causative role in facultative heterochromatin formation (Figures 5 and 6). This hypothesis was confirmed by cloning the *P. citri *HP1-like gene [40] which was found to coincide with *pchet2, *a chromodomain-containing gene identified by Epstein and collaborators in 1992 [55]. The *pchet2 *sequence was used to construct double-stranded interfering RNA that was employed to knockout *pchet2* expression in coccid embryos. The knockout resulted in the inhibition of facultative heterochromatin formation [40]. In fact, the lack of chromocenter development following PCHET2 depletion made it very difficult to distinguish male embryos. The role of PCHET2 was also confirmed in some adult tissues in which the reversion of heterochromatinization occurs. In gut tissues, for example, the loss of chromocenters was accompanied by the dispersion of the PCHET2 signal (Figure 6).

The distribution of the histone modifications K9H3me3 and K20H4me3 in *P. citri *nuclei was also coincident with facultative heterochromatin [38, 39]. Moreover, the immunological detection of these histone modifications in male embryos preceded the appearance of facultative heterochromatin. Significantly, *pchet2 *knockout led to the loss of immunostaining for both K9H3me3 and K20H4me3 [40]. Interestingly, a study on the inactivation of the human X chromosome showed a similar colocalization of HP1 with K9H3me3 and K20H4me3 histone modifications on the inactive X [56]. The K9H3me3-HP1-K20H4me3 pathway is thus an evolutionarily conserved mechanism for epigenetic silencing of large chromosomal domains by facultative heterochromatinization.

The pattern of acetylation of histone H4 (AcH4), a histone modification that has been associated with active chromatin was investigated in *P. citri *by Ferraro and collaborators [57], who found that the male-specific heterochromatic chromocenter is devoid of this modification, as is also the case for the inactive X chromosome in female mammals [58].

Interestingly, all the factors implied in mealybug facultative heterochromatin assembly are already associated with constitutive heterochromatin. The same is true of heterochromatin protein HP2 that was isolated as a constituent of *D. melanogaster *constitutive heterochromatin. Using an antibody against the Drosophila HP2, we demonstrated that it also decorates the *P. citri *male-specific heterochromatin [41].

The imprinting cycle features (see the last paragraphs of Section 1) focused the search for its molecular mechanisms on DNA methylation, whose characteristics (establishment, maintenance, and erasure) fulfilled the requirements of imprinting cycle in mammals [23] (Figure 1). In chromosomal domains, where imprinted genes lie, sequence elements have been identified that are essential to the imprinted gene expression. These “imprinting control elements” (ICEs) are rich in CpG dinucleotides (many correspond to CpG islands), which exhibit parent-of-origin-specific differential DNA methylation. Following fertilisation, allele-specific methylation marks are maintained throughout development and modulate the imprinted differential expression of the alleles [59]. These regions of differential methylation (DMRs) may be either at the boundary between reciprocally imprinted genes or in the promoter of antisense silencing RNAs [60, 61]. In mealybugs, the role of DNA methylation in imprinting was first studied by Scarbrough and collaborators in 1984 [62]. These authors showed the presence of methylated cytosines in the male genome of *P. calceolariae *and measured, by HPLC, the total amount of methylated cytosines in males (0.68 + 0.02%) and females (0.44 + 0.04%) [62]. However, these studies failed to directly correlate DNA methylation and chromosome heterochromatinization. The occurrence of CpG methylation in *P. citri *was confirmed by our group at both the molecular and cytological levels [63]. We showed that the paternally derived chromosomes were hypomethylated at CpG dinucleotides compared to maternal chromosomes in both males, where they were inactivated, and females, where they remained active. This result indicates that in mealybugs, as in mammals, parent-of-origin-specific differential DNA methylation is the molecular signal to imprint chromosomes. However, since in males paternal heterochromatic chromosomes are less extensively methylated than their maternal euchromatic counterparts, we concluded that DNA methylation in mealybugs does not induce genetic inactivation, as it occurs in vertebrates [63]. On the other hand, the lack of a direct correlation between DNA methylation and gene silencing seems to be a common feature in insects (reviewed in [64]).

## 4. The Mealybugs as a Paradigm of Epigenetics

**Figure 7:**
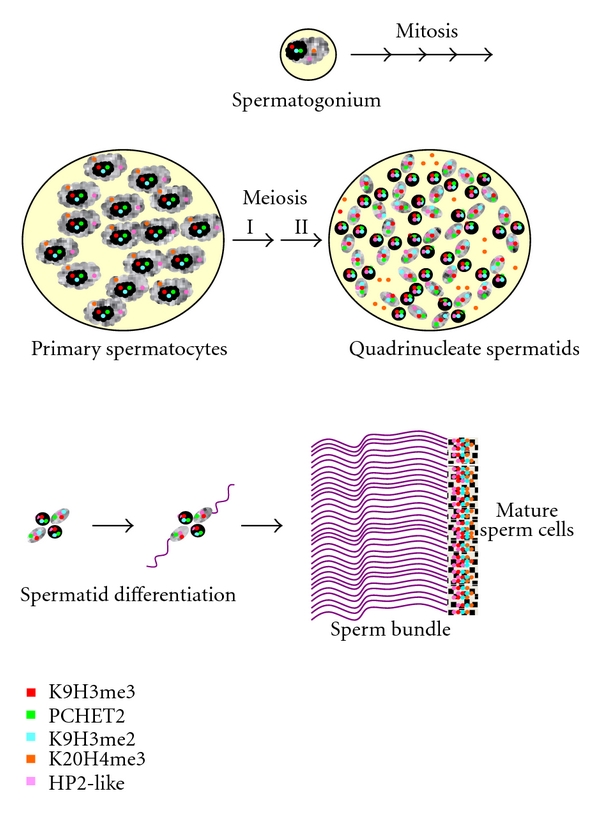
Epigenetic marks during *P. citri *spermatogenesis. A drawing representing the behavior of K9H3me3 (red), PCHET2 (green), K9H3me2 (light blue), K20H4me3 (orange), and of the HP2-like protein (pink), during spermatogenesis in *P. citri*. The HP1-like, PCHET2 is not detectable in sperm heads. According to Koshla et al. [66], sperm chromatin contains also a nuclease resistant fraction (NRC) that is transmitted to the progeny and that can be well considered as a component of the epigenetic machinery.

The reprogramming of the parent-of-origin-specific epigenetic marks during gametogenesis is one of the key features of genomic imprinting. In mealybugs, the chromatin remodeling events that occur during gametogenesis and lead to the facultative heterochromatinization of an entire haploid set of chromosomes in the male progeny were thoroughly scrutinized by immunocytological analysis of male and female gametogenesis (Figure 7) [42]. K9H3me3, K9H3me2, K20H4me3, PCHET2, and HP2-like could not be detected in females from meiosis to mature oocytes, whereas in males, they marked all stages from spermatogonia to spermatids, with a distribution pattern that changed according to cell type. In spermatogonia, for example, whereas K9H3me3, K9H3me2, and PCHET2 were enriched within the heterochromatin, HP2-like and K20H4me3 were found in the euchromatin [42]. However, at the spermatid stage, K9H3me3, K9H3me2, PCHET2, and HP2-like reallocated over both the euchromatin- and the heterochromatin-containing spermatids, which were produced by nonindependent assortment during inverted meiosis. The redistribution of epigenetic signals in spermatids might be related to the establishment of parental imprinting. These results were in agreement with the model proposed by Brahmachari and collaborators [65, 66], who described the reorganization of the male-specific NRC (nuclease-resistant chromatin) [67, 68] during spermatogenesis. These authors found that NRCs were acquired during maturation by sperm nuclei that contained the maternal, originally NRC-free, chromosome set [66]. Following spermiogenesis, PCHET2, the mealybug HP1-like protein was lost from mature sperm, whereas K9H3me3, K9H3me2, K20H4me3, and HP2-like were still detectable, thus ruling out the possibility that PCHET2 could play a role in the imprinting mechanism. Sperm that entered the oocyte possessed distinct K9H3me3 and K9H3me2 signals that were still found in the early pronucleus. Thus, K9H3 di- and trimethylation turned out to be the best candidates for the marks that imprint the paternal chromosomes. Buglia and Ferraro reported that the two euchromatic spermatids originating from a single meiosis were labeled with different levels of K9H3me3 and of C1A9-positive immunostaining, suggesting that the two resulting sperms produced male or female progeny according to the amount of these epigenetic factors [69]. However, the nuclei of quadrinucleate spermatids share a common cytoplasm thus making it unlikely that an enrichment of K9H3me3 in one of the “euchromatic” spermatids could occur independently from the other “euchromatic” spermatid. Accordingly, in our scrutiny of *P. citri *spermatogenesis, we failed to observe any significant difference of labeling between the two euchromatic spermatid nuclei stemming from the same meiosis, with any of the epigenetic factors we tested [42].

Taken as a whole, all these observations suggest that the sex determination of the zygote is very likely dependent upon some unknown factor(s) that is deposited in the cytoplasm of the egg by mother. This scenario is consistent with the studies of Nelson-Rees [70], who showed that the sex-ratio widely fluctuates from female to female and is markedly influenced by the mother's age. Additionally, in insects sex ratio can be affected by different environmental factors acting on parents, like extreme temperature, starvation, and lack of resources [71–76]. In *P. citri *females, various factors, such as population density [77, 78], temperature [70, 79], and mating age [70, 78, 80], were found to influence sex allocation. Ross and collaborators tested three environmental factors (rearing temperature, food deprivation, age of mating) and showed that the effect of high temperature was rather weak, food restriction appeared to be strongly associated with reduced longevity, while older age at mating affected sex allocation, resulting in female-biased sex ratios [81]. The mechanism of this phenomenon is still unclear although PCHET2 and the histone modifications involved in the facultative heterochromatization [10, 28, 37, 40] are thought to be also involved in sex determination [82]. Females might alter the concentration of these proteins in their eggs to modulate the sex ratio of their broods. Along these lines, Buglia and collaborators observed increased concentrations of a C1A9 positive-staining protein in eggs of females that were aged prior to mating [83]. They supposed that these females would produce male-biased offspring (although the sex ratio data were not provided), whilst the opposite effect of maternal ageing prior to mating was observed in other studies [81].

We can hypothesize that the embryo cytoplasm, at blastoderm stage, determines whether the paternal chromosomes, which are marked by DNA hypomethylation [63] and K9H3 methylation [42], will undergo heterochromatinization or not, giving rise to a male or a female embryo, respectively. Given the causative role of PCHET2 in male-specific heterochromatin formation [40], the amount of PCHET2 in the developing embryo may be crucial to steer the embryo toward male or female development. As above reported, facultative heterochromatinization forms in 7th cleavage male embryos as a wave from one pole of the embryo toward the other [10], suggesting a graded distribution of PCHET2 in the embryo. Since PCHET2 could be evidenced neither in the sperm nor in the oocyte [42], its presence in the embryo should be the result of early *de novo *synthesis under the control of the above-mentioned maternal factor(s).

Khosla et al. have also suggested that a unique chromatin organization is a mechanism of genomic imprinting in coccids [65]. The nuclease resistant chromatin (NRC) [67] represents an altered organization of 10% of the paternal genome, not cytologically equivalent to heterochromatin, but perhaps containing the putative centres for facultative heterochromatin nucleation. At the cleavage stage a choice is made between the maintenance or loss of the NRC transmitted with the sperm, leading to a male or female developmental pathway, respectively [65]. The model of Koshla et al. [65] can be well reconciled with our cytological dissection of imprinting marks during spermatogenesis [42]. We can assume, as suggested by Khosla et al., that NRC regions represent chromosome inactivation centers scattered at many loci along the chromosomes. NRCs may be imprinted in the mature sperm by DNA hypomethylation and K9H3 trimethylation marks that then spread to the whole paternal genome. Then, in the cleavage embryo, some maternal factor(s) might regulate the amount of PCHET2, that gradually spreads from one embryo pole to the other. A critical amount of PCHET2 will then determine whether the paternal imprinted chromosomes will become heterochromatic, thus leading to male development, or will remain euchromatic, losing repressive histone modification and NRCs, and eventually leading to female development.

## 5. Perspectives

Based on the characteristics presented in this paper, the phenomenon of imprinted facultative heterochromatinization in mealybugs represents one of the most remarkable examples of epigenetics in eukaryotes.

The mealybug chromosome system offers a very acute tool with which to dissect the phenomenon of facultative heterochromatinization and the mechanisms of parental imprinting. The conservation in mealybugs of almost all the epigenetic mechanisms that act in mammals strongly supports the use of these species as a model for epigenetics. Most epigenetic mechanisms, such as histone modifications and their interplay with the HP1 proteins, show the same functional role in mealybugs as in mammals; others, namely, DNA methylation, exhibit a different involvement in epigenetics.

From the above considerations it appears that a genome-wide approach to map the distribution of epigenetic marks along coccid genome, represents a new challenge for the functional analysis of epigenomes. The epigenetic landscape of the mealybug genome might be useful (i) to determine if there are DNA sequences that act as inactivation centres, scattered along the chromosomes, as suggested by the inactivation of small fragments from irradiated chromosomes; (ii) to highlight the possible role of small RNAs in facultative heterochromatinization and imprinting; (iii) to analyze a specific functional role for the different histone modifications; (iv) to verify the presence and distribution of DNA methylation and its relationship to the histone modifications.
